# Supplementary material for: The relationship between consultation modality and mental health professional type and perceived quality of care and mental health outcomes: A comparison between employed and unemployed individuals
Source: PLOS Ment Health. 2025 Nov 6;2(11):e0000464. doi: 10.1371/journal.pmen.0000464 (PMC12798431; doi:10.1371/journal.pmen.0000464)
Supplement: Attachment — Submitted filename: Response to Reviewers (2).docx [file pmen.0000464.s001.docx]

**Response to Reviewers**

**Additional Editor Comments**

**Comment #1:** The decision to dichotomize both dependent variables (quality of care and perceived mental health) has no rationale provided. Dichotomization may lead to loss of information and statistical power. The authors should explain why this approach was preferred over treating the variables as ordinal or continuous (e.g., using ordinal logistic regression or treating scales as ordered categories).

**Response #1: Thank you for your comment. Our decision to dichotomize both dependent variables was due to practical and conceptual reasons. We found that some of the sub-groups had smaller sample size, which may increase variability when using ordinal logistic regression. We also aimed to distinguish between participants who reported “poor/fair” versus “good/excellent” quality of care, and similarly “negative” versus “positive” mental health, to be more clinically interpretable for policymakers. We included a rational for this in our methods section and indicated in our limitations that dichotomization can reduce variability.**

**Comment #2:** The text does not describe how model fit was assessed (e.g., multicollinearity among predictors checked, if logistic regression assumptions validated, lack of influential outliers). This section is recommended to be improved.

**Response #2: Thank you for bringing this to our attention. We have used the variance inflation factor (VIF) to test multicollinearity. Results showed acceptable ranges (1.00 to 1.38), suggesting no evidence of collinearity issues. We have now included this information in the text.**

**Journal Requirements**

**Comment #1:** Please ensure that your Ethics Statement is available in its entirety at the beginning of your Methods section, under a subheading 'Ethics Statement'. It must include:

1) The name(s) of the Institutional Review Board(s) or Ethics Committee(s)

2) The approval number(s), or a statement that approval was granted by the named board(s)

3) (for human participants/donors) - A statement that formal consent was obtained (must state whether verbal/written) OR the reason consent was not obtained (e.g. anonymity).

NOTE: If child participants, the statement must declare that formal consent was obtained from the parent/guardian.

**Response #1: Thank you for your comment. We have moved the ethics statement to the beginning of the methods section.**

**Comment #2:** In the online submission form, you indicated that “The dataset used and/or analyzed during the current study are available upon request from the corresponding author”.

All PLOS journals now require all data underlying the findings described in their manuscript to be freely available to other researchers, either
1. In a public repository,
2. Within the manuscript itself, or
3. Uploaded as supplementary information.

This policy applies to all data except where public deposition would breach compliance with the protocol approved by your research ethics board. If your data cannot be made publicly available for ethical or legal reasons (e.g., public availability would compromise patient privacy), please explain your reasons by return email and your exemption request will be escalated to the editor for approval. Your exemption request will be handled independently and will not hold up the peer review process but will need to be resolved should your manuscript be accepted for publication. One of the Editorial team will then be in touch if there are any issues.

**Response #2: Thank you for your comment. The dataset used in our study is publicly available through the Odesi database, which is now cited and referenced within the manuscript.**

**Reviewer #1**

**Comment #1:** Title - After reading the manuscript, I understand that this study is observational and not interventional. If the authors agree with me, the word “influence” in the title could be replaced by “relationship.” Also, in the title, the word ‘type’ is missing from the concept of “mental health professional.” I therefore suggest that the title be changed to “The relationship between consultation modality and mental health professional type and perceived quality of care and mental health outcomes: A comparison between employed and unemployed individuals”

**Response #1: Thank you for your suggestion. Yes, we agree that this study is observational and not interventional. Therefore, we have revised the title accordingly.**

**Comment #2:** Abstract - In this sentence (lines 28-30), I suggest saying: “The type of mental health professional consulted and the type of consultation modality may be associated with individuals’ perceptions...”

Lines 30-35, it seems to me that the study aims to examine the relationship between the type of consultation with different mental health professionals and the perceived quality of care and mental health outcomes...

**Response #2: Thank you for your comment. We have revised the sentence accordingly.**

**Comment #3:** Introduction - If I understand correctly, this study focuses explicitly on mental health professionals. In the context in which the study was conducted, are family doctors (also known as general practitioners in some countries) officially included on the list of mental health professionals alongside psychiatrists, psychologists, etc.? If so, I advise mentioning this explicitly and providing references. If not, please clarify their position as mental health professionals in this study or remove them from the list.

For the purpose of the study (lines 141-144), I suggest stating that: This study aims to examine the relationship between the type of consultation (...) with different types of mental health professionals (...) and the quality of care and self-reported mental health among employed and unemployed Canadians.

**Response #3: Thank you for pointing out this concern. We have explicitly mentioned that family doctors are included on the list of mental health professionals and have cited references.**

**Comment #4:** Methods - The study design does not seem very clear. Please clarify it a little.

Some words (e.g., Black, Chinese, Filipino, South Asian) are repeated (see Lines 157-158). Please check if this is an error.

**Response #4: Thank you for noting this. We have explicitly stated the study design in the methods and have corrected the error regarding the repetition of certain words**

**Comment #5:** Results - The results are very well presented. However, in Table 4, although no relationship between variables has been established, the confidence intervals for the two types of mental health professionals (psychiatrists and psychologists) appear to be very wide in the “unemployed” category. Is there a possible explanation for this?

**Response #5: We appreciate your comment. The wide confidence intervals were likely due to low sample size within these subgroups, which increases variability in the estimates. We have acknowledged this as a limitation and recommend that future studies should consider larger number of unemployed individuals to improve precision and allow for a more reliable comparison across the employment groups.**

**Comment #6:** Discussion - In the following statement: “Our study examined the impact of consultation modality (in-person, remote and hybrid) and provider type (family doctor, psychiatrist, psychologist, and social worker) on self-reported quality of care and perceived mental health outcomes, comparing employed and unemployed individuals.” (Lines 252-255), I would suggest deleting the word “impact” and rewriting it as follows: “Our study examined the relationship between consultation modality (...) and provider type (...) and self-reported quality of care and perceived mental health outcomes, comparing employed and unemployed individuals.”

The limitations of the study are very well elaborated. One limitation that could also be discussed is the type of study design used, which does not allow for establishing cause–effect relationships. However, the study strengths are not described. I suggest titling this subsection “Strengths and Limitations” and elaborating on the methodological study strengths.

In the conclusion (lines 424-426), I would suggest stating that: “This study examined the relationship between the type of consultation—whether in-person, remote, or hybrid—with various mental health professionals and self-reported quality of care and perceived mental health among employed and unemployed Canadians.”

**Response #6: Thank you for your suggestions. We have revised the statements in the discussion and conclusion, added an additional limitation and described the study’s strengths within the discussion section.**

**Comment #7:** References - In the body of the manuscript, please harmonize the citation style for references. Two citation styles are currently used: the numerical style [1], [2], [5] and the author-date style: Snoswell et al. (2021), Henry et al. (2020), Li and Nowrouzi-Kia (2024), Jenkins-Guarnieri et al. (2015).

**Response #7: Thank you for noting this. We have harmonized the citation style for the references.**

**Reviewer #2**

**Comment #1:** Need of the review and the methodology adopted in this review should be stated with supporting literatures. A theoretical framework should be added in this paper. The added information would make the paper more clear and scientific for the authors. I recommend this paper for publication with minor edits.

**Response #1: We appreciate your comment. We have added a theoretical framework to the paper and have stated the need for the review and methodology adopted with supporting literature.**

We want to thank all the reviewers for their suggestions. We believe their feedback has improved the quality of the paper.
